# 24-*nor*-ursodeoxycholic acid ameliorates inflammatory response and liver fibrosis in a murine model of hepatic schistosomiasis

**DOI:** 10.1016/j.jhep.2014.11.020

**Published:** 2015-04

**Authors:** Martina Sombetzki, Claudia D. Fuchs, Peter Fickert, Christoph H. Österreicher, Michaela Mueller, Thierry Claudel, Micha Loebermann, Robby Engelmann, Cord Langner, Emine Sahin, Dorothee Schwinge, Nina D. Guenther, Christoph Schramm, Brigitte Mueller-Hilke, Emil C. Reisinger, Michael Trauner

**Affiliations:** 1 Division of Tropical Medicine and Infectious Diseases, Department of Internal Medicine, University of Rostock, Germany; 2 Hans Popper Laboratory of Molecular Hepatology, Division of Gastroenterology and Hepatology, Department of Internal Medicine III, Medical University of Vienna, Austria; 3 Division of Gastroenterology and Hepatology, Department of Internal Medicine, Medical University of Graz, Austria; 4 Institute of Pathology, Medical University Graz, Austria; 5 Institute of Pharmacology, Center for Physiology and Pharmacology, Medical University of Vienna, Austria; 6 Institute of Immunology, University of Rostock, Germany; 7 Institute for Physiology, Center for Physiology and Pharmacology, Medical University of Vienna, Austria; 8 Department of Medicine I, University Medical Center Hamburg-Eppendorf, Hamburg, Germany

**Keywords:** *S. mansoni*, *Schistosoma mansoni*, *nor*UDCA, 24-*nor*-ursodeoxycholic acid, UDCA, ursodeoxycholic acid, Abcb4/Mdr2, canalicular phospholipid export pump/multidrug resistance protein 2, *B. glabrata*, *Biomphalaria glabrata*, ALT, alanine aminotransferase, AP, alkaline phosphatase, BMDC, bone marrow derived dendritic cells, BMDM, bone marrow derived macrophages, HP, hydroxyproline, M-CSF, macrophage colony-stimulating factor, Liver fibrosis, Bile acids, *Schistosoma mansoni* infection, Hepatic granulomas, Anti-inflammatory/anti-fibrotic therapy

## Abstract

**Background & Aims:**

Intrahepatic granuloma formation and fibrosis characterize the pathological features of *Schistosoma mansoni* infection. Based on previously observed substantial anti-fibrotic effects of 24-*nor*-ursodeoxycholic acid (*nor*UDCA) in *Abcb4/Mdr2*^−/−^ mice with cholestatic liver injury and biliary fibrosis, we hypothesized that *nor*UDCA improves inflammation-driven liver fibrosis in *S. mansoni* infection.

**Methods:**

Adult NMRI mice were infected with 50 *S. mansoni* cercariae and after 12 weeks received either *nor*UDCA- or ursodeoxycholic acid (UDCA)-enriched diet (0.5% wt/wt) for 4 weeks. Bile acid effects on liver histology, serum biochemistry, key regulatory cytokines, hepatic hydroxyproline content as well as granuloma formation were compared to naive mice and infected controls. In addition, effects of *nor*UDCA on primary T-cell activation/proliferation and maturation of the antigen-presenting-cells (dendritic cells, macrophages) were determined *in vitro*.

**Results:**

UDCA as well as *nor*UDCA attenuated the inflammatory response in livers of *S. mansoni* infected mice, but exclusively *nor*UDCA changed cellular composition and reduced size of hepatic granulomas as well as TH2-mediated hepatic fibrosis *in vivo*. Moreover, *nor*UDCA affected surface expression level of major histocompatibility complex (MHC) class II of macrophages and dendritic cells as well as activation/proliferation of T-lymphocytes *in vitro*, whereas UDCA had no effect.

**Conclusions:**

This study demonstrates pronounced anti-inflammatory and anti-fibrotic effects of *nor*UDCA compared to UDCA in *S. mansoni* induced liver injury, and indicates that *nor*UDCA directly represses antigen presentation of antigen presenting cells and subsequent T-cell activation *in vitro*. Therefore, *nor*UDCA represents a promising drug for the treatment of this important cause of liver fibrosis.

## Graphical abstract

**Figure:**
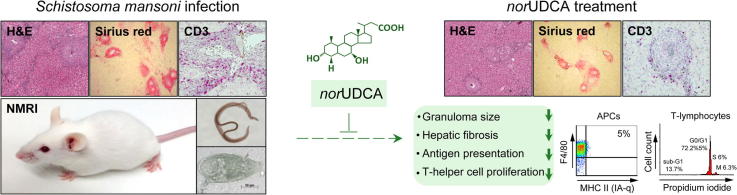

## Introduction

Liver fibrosis represents an overwhelming wound-healing process, characterized by excessive deposition of extracellular matrix, to chronic injury which is frequently driven by inflammation [1], [2]. Soluble factors produced by invading inflammatory cells such as proinflammatory and profibrogenetic cytokines and chemokines play a pivotal role in the activation and transformation process of hepatic stellate cells as major cellular source for extracellular matrix production within the injured liver [3]. *Schistosoma mansoni* (*S. mansoni*) infection demonstrates a leading cause for liver fibrosis, portal hypertension and its sequels include variceal bleeding and ascites [4]. Portal hypertension in *S. mansoni* infected individuals results from increased hepatic resistance to blood flow primarily related to a sustained inflammatory-driven fibrotic process as a consequence of egg-induced granuloma formation [5]. Although antihelminthic therapy is effective to treat *S. mansoni* infection in many patients, portal hypertension and its complications may persist, reflecting the urgent need for novel treatment strategies for *S. mansoni* induced liver fibrosis.

Recently, several novel bile acid derivatives, including side chain-shortened bile acids such as 24-*nor*-ursodeoxycholic acid (*nor*UDCA) [6], were identified as promising new treatment options for common and important diseases such as arteriosclerosis, metabolic syndrome, and liver fibrosis [7], [8], [9], which has fostered a rebirth of bile acid research [10], [11]. Data obtained in *Abcb4/Mdr2*^−/−^ (canalicular phospholipid export pump/multidrug resistance protein 2) mice, representing a well characterized model system for sclerosing cholangitis and biliary type of liver fibrosis, suggest potential direct anti-inflammatory and anti-fibrotic effects of *nor*UDCA [6], [12], [13]. However, since *Abcb4*^−/−^ mice suffer from defective bile formation (i.e., the lack of biliary phospholipid secretion with consecutive toxic bile formation) and *nor*UDCA also has substantial effects on bile formation and composition [14], [15], it remains unclear whether this promising compound is able to exert beneficial mechanisms in non-cholestatic types of liver fibrosis. *S. mansoni* related pathology is mainly induced by cellular immune responses and orchestrated by CD4-positive T-lymphocytes [16], [17]. TH1 dominates the early inflammatory infectious reaction, which shifts towards an egg-driven TH2-reaction later on. Based on the findings of these longitudinal studies, it is suggested that an imbalanced TH1/TH2-response may represent a pivotal trigger for *S. mansoni* induced liver fibrosis [18], [19]. However, the therapeutic potency of novel bile acid analogues such as *nor*UDCA in counteracting the inflammatory and/or fibrotic response in a murine model of hepatic schistosomiasis has not been explored so far.

Therefore, the aim of this study was to explore the therapeutic efficacy of *nor*UDCA *in vivo*, in a mouse model of *S. mansoni* induced liver fibrosis, compared to the effectiveness of its mother compound UDCA.

## Materials and methods

### *S. mansoni* mouse model and infection

Six- to eight-week-old NMRI mice (Charles River Laboratories, Germany) were housed in an animal facility with a 12:12 h light/dark cycle, with *ad libitum* water and free access to standard chow (SSNIFF, Soest, Netherlands). The experimental protocols were performed according to the German animal protection law and approved by the local animal care and use committee. Schistosomal cercariae were generated in a Brazilian *S. mansoni* strain held in a cycle with NMRI mice and *Biomphalaria glabrata* (*B. glabrata*) water snails (Puerto Rico) [20]. *S. mansoni* cercariae were obtained by mass shedding of 5–10 infected *B. glabrata* after light exposure. Mice were exposed to parasites for 90 min by sitting in a water bath enriched with approximately 50 *S. mansoni* cercariae. Infection grade was estimated by determination of hepatic granuloma count in hematoxylin/eosin-stained liver slices using a conventional microscope (magnification 100×).

### Bile acid treatment

Twelve weeks following *S. mansoni* infection, a time point when liver fibrosis is already fully established, mice were fed either standard chow (control, n = 7), 0.5% (wt/wt) *nor*UDCA-supplemented diet (*nor*UDCA, n = 14) or 0.5% (wt/wt) UDCA-supplemented diet (UDCA, n = 12) for four weeks. In addition, naive mice (naive, n = 7) on standard chow were studied. Thereafter, mice were euthanized and liver samples and sera were processed as described previously in detail [21].

### Serum analysis

Enzymatic assays for detection of serum alanine aminotransferase (ALT), alkaline phosphatase (AP) and total serum bile acid levels were performed and analyzed using a cobas® 6000 analyzer (Roche Diagnostics, Mannheim, Germany).

### Liver histology and hepatic hydroxyproline measurement

One part of the right lobe of each liver (lobe 3) was fixed in 4% neutral buffered formaldehyde solution and embedded in paraffin. Four-μm thin sections were stained with hematoxylin/eosin (H&E) and Sirius Red (SR) for detection of collagen fibers. Morphometric analysis of granuloma size was performed using ImageJ software (v1.47v; National Institute of Health). For biochemical quantification of liver fibrosis, hepatic hydroxyproline (HP) content was determined as described previously [6].

### Statistical analysis

Statistical analysis was performed using SPSS (Release 14.0, 2005, SPSS Inc., Chicago, IL). For single time point comparison of two groups the Student’s *t*-test or, where appropriate, the non-parametric Mann–Whitney U-test was used. The number of animals per group was as follows: naive, n = 7; control, n = 7; UDCA, n = 12; *nor*UDCA, n = 14. A *p* value of less than 0.05 was considered significant.

Additional Materials and methods are provided in the Supplementary material section.

## Results

### NorUDCA treatment improves liver histology and reduces granuloma size in hepatic schistosomiasis without any antihelminthic effect

**Fig. 1:**
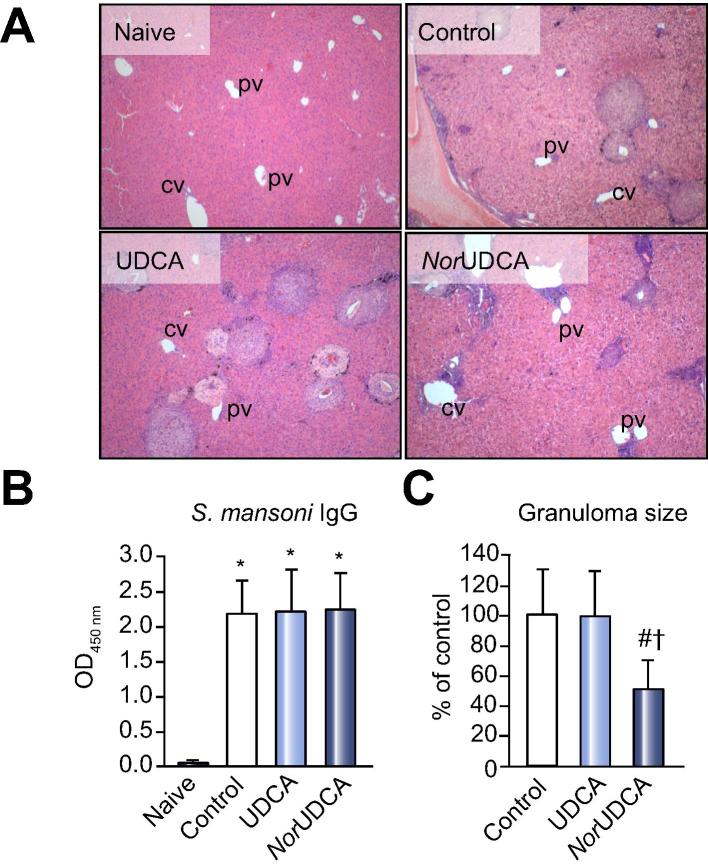
***Nor*UDCA ameliorates liver histology of chronically *Schistosoma mansoni* infected NMRI mice.** (A) Representative liver histology images (HE; original magnification 100×), of female NMRI mice, 16 weeks after infection with 50 *S. mansoni* cercariae, receiving control diet and UDCA (0.5% wt/wt) or *nor*UDCA (0.5% wt/wt) enriched diet for 4 weeks, are shown (original magnification 100×). In addition, a naive (uninfected) group was studied. (B) Infection with 50 *S. mansoni* cercariae resulted in uniformly infection levels in all infected groups, confirmed by antibody detection directed against parasite eggs. (C) Morphometric analysis of granuloma diameter revealed a significant reduction of granuloma size after *nor*UDCA treatment compared to control and UDCA group. ^∗^*p* <0.05 (*vs.* naive), ^#^*p* <0.05 (*vs.* control), ^†^*p* <0.05 (*vs.* UDCA).

Infection with *S. mansoni* cercariae was uniformly accomplished in all experimental groups, proven microscopically (by a board certified expert pathologist, C.L.), by determination of granuloma count/per low power field (control: 7.6 ± 2.3; UDCA: 5.5 ± 1; *nor*UDCA: 6 ± 1.5) and via quantification of antibodies against parasite eggs (Fig. 1A and B). Morphometric analysis of liver slices demonstrated a significant reduction (up to 50%) of the granuloma size after *nor*UDCA treatment, compared to UDCA and control (% of control: UDCA, 98 and *nor*UDCA, 51) (Fig. 1C). Serum parameters for hepatocellular injury (ALT) remained unchanged 16 weeks following infection, while bile acid feeding resulted in significantly higher levels of serum AP levels compared to naive and control mice (Table 1). Notably, the local ductular reaction (in response to granuloma formation), reflected by keratin 19 (K19) staining, was enhanced after UDCA treatment but attenuated after *nor*UDCA treatment (Supplementary Fig. 1).

**Table 1:** **Serum liver biochemistry.**

Serum biochemistry of naive mice (healthy; n = 7) *vs.* control (infected, untreated; n = 7), UDCA (infected, UDCA-treated, n = 12) and *nor*UDCA (infected, *nor*UDCA-treated, n = 14). Results are given as mean ± SD.

ALT, alanine aminotransferase; AP, alkaline phosphatase; BA, bile acids.

^∗^*p* <0.05 (*vs.* naive), ^#^*p* <0.05 (*vs.* control), ^†^*p* <0.05 (*vs.* UDCA).

Furthermore, *in vitro* studies were designed to explore potential direct antihelminthic effects of bile acids. All isolated adult *S. mansoni* worms died within 6 h by control incubation with Praziquantel. Monitoring of bile acid treated groups over 5 days showed no antihelminthic effects of *nor*UDCA or UDCA (% of vital worms after 5 days of incubation: Praziquantel, 0; control, 95.8; UDCA, 93.3; *nor*UDCA, 95.5).

### UDCA and norUDCA treatment attenuates inflammatory reaction in hepatic schistosomiasis

**Fig. 2:**
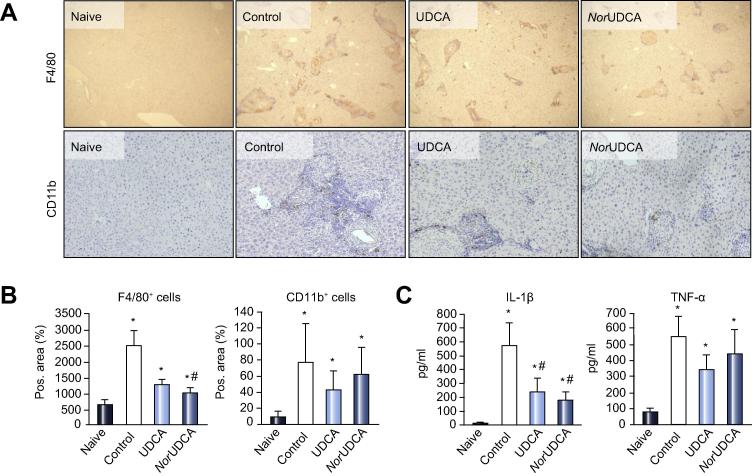
***Nor*UDCA and UDCA suppress inflammatory cell infiltration and inflammatory response.** (A) Representative immunohistochemistry for F4/80^+^ (original magnification 40×) and CD11b^+^ (original magnification 100×) cells within liver specimens of all subjects is shown. *Nor*UDCA treatment leads to a pronounced disaggregation of inflammatory cell infiltrate. (B) Quantification of F4/80^+^ macrophages (ImageJ analysis) revealed a significant reduction of macrophages within *S. mansoni* granulomas after *nor*UDCA treatment while CD11b^+^ cell count did not show bile acid specific differences. (C) Serum levels of TH1 cytokines IL-1 beta and TNF-alpha were measured by ELISA. Both cytokines were significantly elevated after *S. mansoni* infection. Four-week feeding with UDCA and *nor*UDCA treatment counteracted this elevation for IL-1 beta but not for TNF-alpha. Protein values were calculated by standard curve concentrations of recombinant IL-1 beta and TNF-alpha. Concentrations were expressed as means ± SD of duplicates. ^∗^*p* <0.05 (*vs.* naive), ^#^*p* <0.05 (*vs.* control).

Immunohistochemical analysis of liver slices revealed a substantial accumulation of F4/80^+^ and CD11b^+^ monocytes/macrophages and granulocytes around tissue entrapped eggs, in untreated controls compared to naive mice (Fig. 2A). Treatment with both bile acids led to a significant reduction of F4/80^+^ (but not CD11b^+^) cells compared to the untreated control (Fig. 2B).

**Fig. 3:**
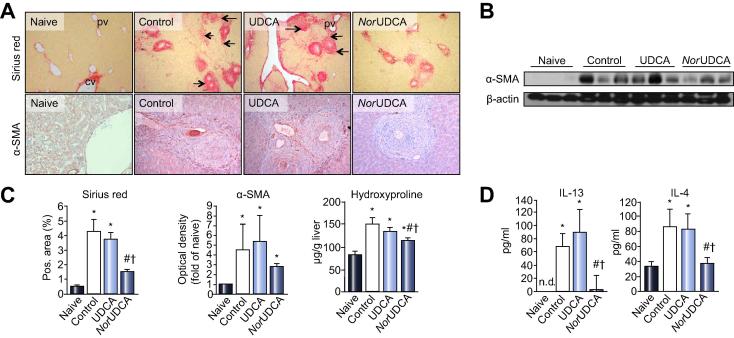
***Nor*UDCA significantly reduces hepatic fibrosis in chronically infected *Schistosoma mansoni* mice.** (A) Representative liver histology (Sirius Red staining, SR; original magnification 100×), of female NMRI mice 16 weeks after infection with 50 *S. mansoni* cercariae, receiving control diet and UDCA (0.5% wt/wt) or *nor*UDCA (0.5% wt/wt) enriched diet for 4 weeks, is shown (original magnification 100×). Hepatic fibrosis with pronounced expansion of connective tissue between hepatic granulomas was most conspicuous in the control and UDCA group, and was reduced after *nor*UDCA feeding. Immunohistochemistry for alpha-SMA (original magnification 200×) revealed a pronounced number of positive cells within fibrotic areas and around egg granulomas in non-treated (control) and UDCA fed mice. Reduced numbers of alpha-SMA^+^ cells under *nor*UDCA treatment can be observed. (B) Representative Western blot for alpha-SMA and beta-actin (as loading control). (C) Fibrosis and alpha-SMA levels were further analyzed by computerized quantification of SR positive areas of liver sections, Western blot densitometry, and hydroxyproline measurements. (D) Serum levels of profibrotic TH2 cytokines IL-13 and IL-4 were measured by ELISA. Both cytokines were significantly elevated 16 weeks following *S. mansoni* infection. *Nor*UDCA (but not UDCA) feeding for 4 weeks resulted in a significant reduction of IL-13 and IL-4 serum level. Protein values were calculated by standard curve concentrations of recombinant IL-13 and IL-4. Protein concentrations were expressed as means ± SD of duplicates. ^∗^*p* <0.05 (*vs.* naive), ^#^*p* <0.05 (*vs.* control), ^†^*p* <0.05 (*vs.* UDCA).

Serum levels of IL-1 beta and TNF-alpha were significantly increased 16 weeks following *S. mansoni* infection in control mice compared to the naive group. Both bile acids significantly antagonized this effect for IL-1 beta (Fig. 2C). This goes in line with mRNA expression profile of classical proinflammatory genes (Supplementary Table 2). Both bile acids tended to reduce classical inflammation markers to similar degrees, although this was not reflected by fibrosis with UDCA. In addition, markers for alternatively activated macrophages were assessed. Arg1 expression profile displayed a significant reduction following *S. mansoni* infection (control) compared to naive mice, whereas UDCA and *nor*UDCA only tended to reduce Arg1. Notably, we observed a significant reduction of Retnla expression after *nor*UDCA treatment comparable to Retnla levels of naive mice. Both bile acids exert similar effects on classical proinflammatory genes whereas only *nor*UDCA affected alternatively activated macrophages in case of Retnla. Moreover, *nor*UDCA (but not UDCA) reduced interleukin (IL)-13 and -4 serum levels (Fig. 3D).

### NorUDCA significantly reduces liver fibrosis in hepatic schistosomiasis

SR-stained liver slices of control and UDCA-treated mice showed expanding fibrosis with pronounced portal-portal bridging that was significantly reduced in response to *nor*UDCA treatment (Fig. 3A). This was supported by a reduction of alpha-SMA^+^ cells on liver slices (Fig. 3A) and reduced alpha-SMA protein levels (Fig. 3B) as well as hepatic hydroxyproline (HP) levels, as markers for biochemical quantification of liver fibrosis by *nor*UDCA (Fig. 3C). IL-13 and -4 serum protein levels were determined since these cytokines have a regulatory importance in TH2 driven liver fibrosis. Serum levels of both profibrotic cytokines were significantly reduced after *nor*UDCA treatment compared to the control and UDCA groups (Fig. 3D). In addition, as shown in Supplementary Table 2, *nor*UDCA reduced TGF-beta expression. Interestingly, infected mice displayed a characteristic increase of matrix metalloproteinase-2 (*MMP-2*) and tissue inhibitor of metalloproteinase (*TIMP-1*) mRNA expression levels, which was reduced by both bile acids treatments (Supplementary Table 2). Notably, the *TIMP-1* to *MMP-2* ratio was reduced only by *nor*UDCA (but not UDCA), which may reflect a beneficial shift favouring matrix degradation (Supplementary Table 3).

Taken together, these data indicate that *nor*UDCA diminished development and ameliorated parameters of hepatic fibrosis, whereas UDCA had no effect on liver fibrosis, in this model system.

### NorUDCA treatment reduces content of inflammatory cell infiltrate and changes cell composition of hepatic *S. mansoni* granulomas

**Fig. 4:**
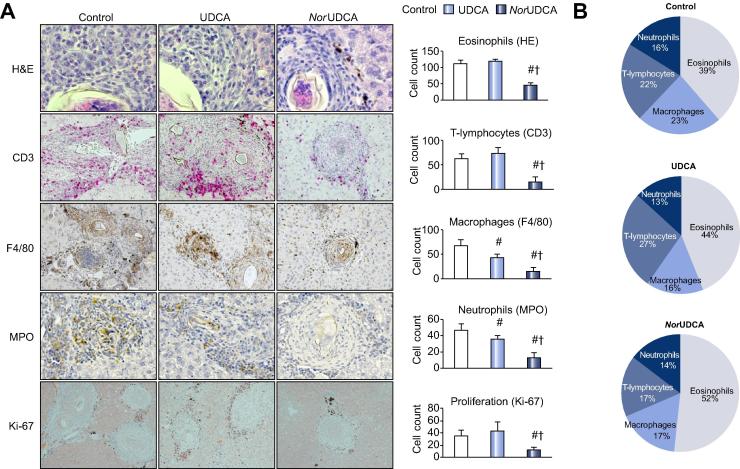
***Nor*UDCA treatment reduces content of inflammatory cell infiltrate and changes cell composition of hepatic *Schistosoma mansoni* granulomas.** Immunohistochemistry on liver slices was performed to characterize cell composition of hepatic *S. mansoni* granulomas with or without bile acid treatment. (A) Representative images for H&E (eosinophils, original magnification 630×), CD3 (T-lymphocytes, original magnification 400×), F4/80 (macrophages, original magnification 200×), and MPO (neutrophils, original magnification 400×) are shown. IHC for Ki-67 (cellular proliferation, original magnification 100×) revealed a significant reduction of dividing cell activity within the *nor*UDCA group compared to UDCA and control. Respective cell numbers were determined microscopically by counting positive cells in 20 hepatic granulomas (hg) per liver slide of each mouse (control: n(hg) = 140; UDCA: n(hg) = 240; *nor*UDCA: n(hg) = 280). Schistosomal hemozoin pigment (brownish pigment) within portal tract and parenchymal interface can be observed. (B) Quantification of cell populations within hepatic granulomas revealed a significant change in cell composition towards a lower content of CD3^+^ T-lymphocytes. ^#^*p* <0.05 (*vs.* control), ^†^*p* <0.05 (*vs.* UDCA).

Since *nor*UDCA treatment resulted in significantly reduced granuloma size, immunohistochemistry was performed to analyze potential changes in cell composition of hepatic *S. mansoni* granulomas after bile acid treatment. Cell numbers of macrophages (F4/80), T-lymphocytes (CD3), neutrophils (MPO), and eosinophils (HE-staining) were determined microscopically, by counting positive cells in 20 hepatic granulomas per liver slide of each mouse. In *nor*UDCA treated mice, all analyzed cell types were significantly reduced compared to untreated control and UDCA. In addition, the relative cellular composition of inflammatory cells within hepatic granulomas of this group was changed with a significant reduction of CD3^+^ T-lymphocytes. IHC for Ki-67 revealed reduced cell proliferation within hepatic granulomas of *nor*UDCA treated mice (Fig. 4A and B).

### NorUDCA impairs surface expression of major histocompatibility complex (MHC) class II of antigen presenting cells and blocks T-cell-receptor dependent and independent activation/proliferation without inducing apoptosis

**Fig. 5:**
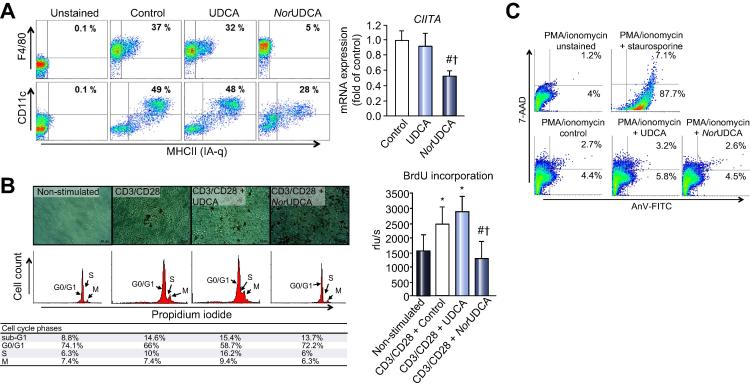
***Nor*UDCA significantly reduces expression of major histocompatibility complex class II molecules (MHC II) on bone marrow-derived macrophages (BMDM) and dendritic cells (BMDC) and inhibits proliferation of primary CD4^+^ T-lymphocytes.** (A) Haematopoietic stem cells of uninfected female NMRI mice were incubated with either UDCA (50 μM) or *nor*UDCA (500 μM), according to their achievable intrahepatic concentrations *in vivo*, and then analyzed for the two directions of APC lineages by flow cytometry. *Nor*UDCA significantly decreased percentage of surface MHC class II expression on APCs, demonstrated by surface double-staining with specific markers for BMDMs (F4/80) and BMDCs (CD11c). mRNA expression level of the key MHC class II regulator *CIITA* was significantly downregulated after *nor*UDCA incubation, in contrast to UDCA and control. (B) CD4^+^ T-lymphocytes, isolated from chronically *S. mansoni* infected NMRI mice, were stimulated with CD3/CD28 dynabeads. *Nor*UDCA significantly inhibited proliferation of T-lymphocytes, confirmed by BrdU incorporation. Stimulated, *nor*UDCA incubated T-lymphocytes did not enter cell cycle interphase (propidium iodide staining); whereas control and UDCA treated cells started to proliferate following stimulation. (C) T-lymphocytes from mesenteric lymph nodes of *S. mansoni* infected mice were isolated and restimulated with PMA/Ionomycin, and incubated with staurosporine (1 μM, positive control), UDCA (50 μM) or *nor*UDCA (500 μM) for 4 h. AnV-FITC (apoptosis) positive cells were most apparent after incubation with staurosporine (95%), while bile acid sublimation (UDCA and *nor*UDCA) did not induce apoptosis. For validation of the method and reproducibility of the results, the experiments were repeated in two independent series. ^∗^*p* <0.05 (*vs.* unstimulated control), ^#^*p* <0.05 (*vs.* control), ^†^*p* <0.05 (*vs.* UDCA).

Populations of double positive bone marrow derived macrophages (BMDM: F4/80^+^/MHC II^+^) and dendritic cells (BMDC: CD11c^+^/MHC II^+^) were analyzed by flow cytometry. Comparable amounts of double positive BMDMs were generated in control and UDCA-treated cells, whereas *nor*UDCA significantly reduced the amount of double positive BMDMs (Fig. 5A). The same pattern was observed in BMDCs. Only half of CD11c^+^ cells present an MHC II signal on their surface. This was also reflected by significantly reduced CIITA expression levels in BMDMs, after *nor*UDCA incubation (Fig. 5A).

CD3/CD28 activation of CD4^+^ primary mouse T-lymphocytes led to a strong proliferation in controls and UDCA-treated cells, demonstrated by BrdU incorporation, while *nor*UDCA completely repressed proliferation of CD3/CD28 (receptor dependent) as well as PMA/ionomycin (receptor independent) activated CD4^+^ T-lymphocytes (Fig. 5B). This has been verified by reduced intracellular CFSE concentration due to cell division activity (Supplementary Fig. 2). Moreover, *nor*UDCA treated T-lymphocytes did not enter interphase of cell cycle compared to control and UDCA (Fig. 5B). To investigate potential apoptotic effects of *nor*UDCA, annexin-V/7-AAD staining was performed. Neither UDCA nor *nor*UDCA induced programmed cell death (Fig. 5C).

Taken together, these data indicate that *nor*UDCA, in contrast to UDCA, directly impairs antigen presentation of APCs, by CIITA regulated suppression of MHC II expression [20] *in vitro*, and further indicate anti-proliferative properties of *nor*UDCA in regard to inhibited activation (proliferation) of T-lymphocytes.

## Discussion

This study aimed at investigating the therapeutic properties and potential direct anti-inflammatory and anti-fibrotic mechanisms of the novel bile acid *nor*UDCA, in an inflammation-mediated model of liver fibrosis without cholestasis [21]. We have chosen its chemical mother compound UDCA as a clinical comparator.

Previous experiments, using cholestatic *Abcb4*^−/−^ mice with sclerosing cholangitis and biliary type of liver fibrosis, have demonstrated anti-fibrotic effects of *nor*UDCA [6] and, to a lesser degree, UDCA; the latter even aggravated liver injury at high doses [22]. Since both drugs are anti-cholestatic and previously observed anti-fibrotic effects may at least in part be related to these mechanisms, we searched for an alternative – preferentially non-cholestatic – model for liver fibrosis, to further discriminate the differential effects of both interesting therapeutic bile acids. *S. mansoni* infected NMRI mice develop a robust and sustained hepatic fibrosis without significantly elevated serum parameters for hepatocellular injury [21]. Proliferation of bile ducts was observed in areas of hepatic granulomas, while “granuloma free” area was completely unremarkable. Notably, the local ductular reaction in response to granulomas was even enhanced after UDCA treatment but declined after *nor*UDCA treatment. In areas of hepatic granulomas, where pre-existing bile ducts may be compressed, choleretic UDCA could provoke a local aggravation of the biliary reaction, as observed under more pronounced cholestatic conditions (e.g., *Abcb4/Mdr2*^−/−^ mice and bile duct ligation), by enhancing biliary pressure and subsequent bile infarcts while *nor*UDCA was beneficial [22], [23]. However, in the current *S. mansoni* mouse model, UDCA did not aggravate hepatic injury or inflammation compared to untreated control, as reflected by unchanged ALT levels and even improved inflammatory markers. Although, *nor*UDCA is also a potent choleretic, the associated induction of bicarbonate rich choleresis as a result of cholehepatic shunting [23], and its antiproliferative properties [24], may explain its beneficial effects on ductular reaction. In this regard, it is also important to note that elevated serum bile acid levels, as observed in the current study in *nor*UDCA-fed *S. mansoni*-infected mice, may be attributed to the high bioavailability of cholehepatic-shunting *nor*UDCA, rather than reflecting a cholestatic condition. This is related to the relative conjugation resistance of *nor*UDCA [13], [15]. As such, the reabsorption of secreted *nor*UDCA by cholangiocytes leads to high serum and intrahepatic *nor*UDCA concentrations [6], [25]. As a possible consequence, the observed beneficial anti-fibrotic and anti-inflammatory effects of *nor*UDCA *in vivo* could, at least in part, be related to direct antihelminthic actions of *nor*UDCA. However, the reduction of granuloma size in the presence of unchanged absolute granuloma numbers and unchanged survival of isolated worms and eggs *in vitro* argues against this interesting option.

The severity of *S. mansoni* pathology depends at least on the balance of TH1 and TH2 mediated responses, and is mostly related to a dysbalance of matrix degradation and its inhibition [26]. Herein we demonstrate a significantly shifted ratio of MMP-2 and TIMP-1 towards MMP-2 after *nor*UDCA treatment, and markedly reduced inflammatory response in *S. mansoni* infected mice, after UDCA and *nor*UDCA feeding. However, only *nor*UDCA treatment led to a reduced granuloma size with secondary beneficial effects on TH2-cytokine (IL-13 and -4) driven hepatic fibrosis. In hepatic schistosomiasis, inflammation (TH1) and fibrosis (TH2) are differentially regulated since it is known that IL-13 affects fibrosis directly in a TGF-beta independent manner [27], and *IL-13^−/−^* mice failed to develop an adequate fibrotic response to *S. mansoni* egg antigens [28]. In our study, we demonstrated comparable effects of UDCA and *nor*UDCA on proinflammatory cytokines, but without any beneficial effects on hepatic fibrosis in UDCA treated mice, possibly indicating a minor relevance of these markers for *S. mansoni* related disease progression. The specific anti-fibrotic effect of *nor*UDCA in this model may be linked to mechanisms directed against TH2 response. This concept is further supported by *nor*UDCA effect on Retnla, presenting a marker for alternatively activated macrophages and negative regulator of TH2-response [29], [30]. Notably, we observed a significant reduction of Retnla expression after *nor*UDCA treatment, comparable to Retnla levels of naive mice. Unfortunately, little is known about Retnla function in TH2 immunity and affected cell types, but its regulation is controlled by IL-4/IL-13 and STAT6 [31], [32]. We demonstrated reduced serum levels of both TH2 cytokines (IL-13 and -4) accompanied by reduced numbers of CD3^+^ and Ki-67^+^ cells in granulomas of the *nor*UDCA (but not UDCA) group. The relevance of local expansion of activated T-lymphocytes for granuloma formation and controlling of inflammatory processes is still unclear [33]. However, our analysis revealed a significantly changed percentage distribution of cellular composition of hepatic granulomas after *nor*UDCA treatment accompanied by reduced granuloma size, declined formation of fibrotic septa, and reduced hepatic fibrosis. In addition, we could demonstrate a reduction of Ki-67^+^ cells at the edges of granulomas, where CD3^+^ cells are located; suggesting that local proliferation of T-lymphocytes may be restrained after *nor*UDCA treatment. This is in line with our *in vitro* settings of restimulated T-lymphocytes with anti-CD3/CD28 coupled beads and receptor independent stimulation with PMA/Ionomycin that was completely detained after *nor*UDCA incubation without inducing apoptosis in these cells.

In addition, a strongly reduced expression of MHC II molecules on BMDMs as well as BMDCs after incubation with *nor*UDCA could in turn provide an explanation for a reduced antigen presentation and subsequent T-cell activation observed with *nor*UDCA. Noteworthy, in our setting UDCA did not show any effects on maturation of APCs and MHC II expression. However, UDCA is able to directly reduce MHC II expression on biliary epithelium and hepatocytes by activating the glucocorticoid receptor, followed by an inhibited IFN-γ-mediated MHC II activation [34]. Together with our observation on alternatively activated macrophages, we suggest that the UDCA effect might be restricted to the classical activation cascade of macrophages and is lost upon alternatively IL-4/IL-13 activated macrophages, within a TH2 milieu [35], [36]. Further characterization of the APC/T-cell interplay is clearly required to obtain additional insights into the anti-inflammatory and immune modulatory mechanisms of *nor*UDCA activity and the interactions with the complex inflammation process of *S. mansoni* infection.

In summary, this study demonstrates: (i) beneficial effects of *nor*UDCA on granuloma size and hepatic fibrosis, (ii) anti-inflammatory properties of *nor*UDCA directed to MHC class II protein expression on dendritic cells and macrophages, and (iii) direct anti-fibrotic effects of *nor*UDCA by reduced T-lymphocyte proliferation and finally reduced serum levels of IL-13 and IL-4. This potentiality may qualify *nor*UDCA as a promising drug for non-cholestatic, inflammation-driven liver fibrosis.

## Financial support

The study was supported by grants P-19118 and F3517-B20 from the Austrian Science Foundation and a GEN-AU project grant from the Austrian Ministry for Science to Michael Trauner. Martina Sombetzki was supported by a Ph.D. scholarship (Landesgraduiertenförderung des Landes Mecklenburg-Vorpommern, Germany).

## Conflict of interest

PF and MT received a research grant from the Dr. Falk Pharma Gmbh, Freiburg, Germany and the authors received *nor*UDCA from Falk for this study. The Medical University of Graz has filed patents (WO 2006/119803 A1 and WO 2009/013334) on the medical use of *nor*UDCA and PF and MT are listed as co-inventors.

## Authors’ contributions

Substantial contributions to the conception and design; or the acquisition, analysis, or interpretation of the data: MS, CDF, PF, RE, DS, NDG, CO, MM, TC, ES; MT.

Drafting of the article or critical revision for important intellectual content: MS, MT, PF, ML, ECR, CL.

Final approval of the version to be published: MT, ECR, BMH, CS.

Agreement to be accountable for all aspects of the work in ensuring that questions related to the accuracy or integrity of any part of the article are appropriately investigated and resolved: MT, ECR.
