# Supplementary material for: 24-nor-ursodeoxycholic acid ameliorates inflammatory response and liver fibrosis in a murine model of hepatic schistosomiasis
Source: J Hepatol. 2015 Apr;62(4):871–8. doi: 10.1016/j.jhep.2014.11.020 (PMC4368108; doi:10.1016/j.jhep.2014.11.020)
Supplement: Supplementary data [file mmc1.pdf]

Supplementary data to:

24-*nor*-ursodeoxycholic acid ameliorates inflammatory response and liver fibrosis in a murine model of hepatic schistosomiasis

Martina Sombetzki, Claudia D. Fuchs, Peter Fickert, Christoph H. Öesterreicher, Michaela Mueller, Thierry Claudel, Micha Loebermann, Robby Engelmann, Cord Langner, Emine Sahin, Dorothee Schwinge, Nina D. Guenther, Christoph Schramm, Brigitte Mueller-Hilke, Emil C. Reisinger, Michael Trauner.

Table of Contents

Supplementary data to: 1

Materials and Methods 2

Enzyme linked immunosorbent assay for Interleukin-13, Interleukin-4, Tumor necrosis factor-alpha, Interleukin-1 beta and Schistosoma mansoni egg antigen. 2

Generation of bone marrow-derived macrophages (BMDM) and dendritic cells (BMDC). 2

Magnetic-activated cell sorting purification (MACS) of $CD4^{+}$T-cell subsets and expansion. 3

Flow cytometry (fluorescence activated cell sorting, FACS). 3

Preparation of adult worms 4

Annexin-V-FITC/7AAD apoptosis assay. 4

CFSE Cell proliferation assay. 5

RNA isolation, reverse-transcription and TaqMan real-Time polymerase chain reaction. 5

Immunohistochemistry for F4/80, CD11b, CD3, MPO, Ki-67, K19 and alpha-SMA. 6

Western blotting for alpha-smooth muscle actin. 6

Supplementary figures and Supplementary figure legends 7

Supplementary Figure 1. 7

Supplementary Figure 2. 8

Supplementary Tables 9

Supplementary Table 1. Antibodies for Immunohistochemistry used in this study. 9

Supplementary Table 2. mRNA expression pattern under various experimental conditions (Sybr Green analysis). 10

Supplementary Table 3. Ratio of mRNA levels of major matrix degradation enzyme (MMP-2) and its inhibitor (TIMP-1) under various experimental conditions (Sybr Green analysis). 11

Supplementary References 12

Materials and Methods

Enzyme linked immunosorbent assay for Interleukin-13, Interleukin-4, Tumor necrosis factor-alpha, Interleukin-1 beta and Schistosoma mansoni egg antigen.

Enzyme linked immunosorbent assays (ELISA) for interleukin (IL)-1 beta, -4, -13, tumor necrosis factor-alpha (TNF-alpha) and *Schistosoma mansoni* egg antigen were performed using specific ELISA-kits (IL-4 and 13, Promokine, Heidelberg, Germany; IL-1 beta and TNF-alpha, eBioscience, San Diego, USA; *Schistosoma mansoni* egg antigen ELISA, Viramed, Planegg, Germany). According to the manufacturers' guidelines standard curves were prepared to calculate protein concentrations from standard optical densities versus concentrations. Protein levels were expressed as pg/ml ± standard deviation (SD) of two technical replicates.

Generation of bone marrow-derived macrophages (BMDM) and dendritic cells (BMDC).

BMDMs and BMDCs were isolated and cultured as previously described [Supplementary Ref. 36]. To test the effects of bile acids on cell differentiation, medium was supplemented with either UDCA (50 µM) or *nor*UDCA (500 µM). Bile acid concentrations were selected according to their reported intrahepatic concentrations reflecting differences in cholehepatic shunting with intrahepatic enrichment [6] to simulate *in vivo* conditions. On day 7 matured BMDCs and matured BMDMs were harvested and subsequently phenotyped by flow cytometry (marker for BMDMs: F4/80-FITC, BM8, Abcam, Cambridge, UK; marker for BMDCs: CD11c-FITC, HL3, BD, Heidelberg, Germany; marker for MHC II: (I-Aq)-Alexa 647, KH116, BioLegend, San Diego, CA).

Magnetic-activated cell sorting purification (MACS) of $CD4^+$T-cell subsets and expansion.

Twelve weeks following *S.m.* infection mice were sacrificed by cervical dislocation. Mesenteric lymph nodes (mLN) were dissected and gently forced through a cell-strainer (70 µm). Cells were suspended in RPMI with 10% (v/v) FCS, 1% penicillin/streptomycin and 0.5 µM β-mercaptoethanol). $CD4^+$ cells were isolated by positive selection with directly conjugated anti-CD4 magnetic microbeads (Miltenyi Biotec, Bergisch Gladbach, Germany) and routinely showed more than 93% purity for $CD4^+$ cells by flow cytometry analysis. For T-cell receptor dependent restimulation positive selected $CD4^+$ lymphocytes were grown in RPMI-medium (described above) supplemented with 50 units/mL rIL-2, 25 µl/1x10^6cells CD3/CD28 microbeads (Dynal, Lake Success, NY) and with bile acids (50 µM UDCA and 500 µM *nor*UDCA) or without bile acids (control) for 5 days. 24h prior assessment of T-cell proliferation bromodeoxyuridine-labeling (BrdU-labeling) solution was added. BrdU incorporation was measured according the manufacturer's instructions (Roche Applied Systems, Mannheim, Germany). For T-cell receptor independent restimulation we used a cell stimulation cocktail on basis of 12-myristate 13-acetate (PMA) and Ionomycin (affymetrix ebioscience, Frankfurt/Main, Germany). To verify BrdU Assay CellTrace™ CFSE Cell Proliferation Assay (Invitrogen Life Technologies, Darmstadt, Germany) was performed according to the manufacturer's instructions. To ascertain any potential bile acid effects on cell cycle we performed a Nicoletti Assay (FACS analysis) using Propidium iodide (20 µg/ml) staining following RNA digestion (RNase A, 10µg/ml).

Flow cytometry (fluorescence activated cell sorting, FACS).

Antigen presenting cells (APC) were surface stained with specific antibodies for F4/80-FITC (BM8, Abcam, Cambridge, UK), CD11b/PE (M1/70, BD, Heidelberg,

Germany), CD11c/FITC (HL3, BD, Heidelberg, Germany) and MHC II (I-Aq)-Alexa 647 (KH116, BioLegend, San Diego, CA) for 15 min at 4°C, and then washed in FACS-buffer (PBS pH 7.4, 0.5% BSA, 0.1% NaN3). Events were collected on FACS Calibur™ (BD Biosciences) driven by CellQuest pro software (v3.3, BD, Heidelberg, Germany). Respective cell populations were analyzed using WinMDI (v2.9) software.

Preparation of adult worms.

To determine a potential direct antihelminthic impact of bile acids, UDCA and *nor*UDCA were tested in an *in vitro* assay with isolated adult *S.m.* worms in comparison to Praziquantel (standard antihelminthic drug). The isolation of adult *S.m.* stages was performed using perfusion technique described elsewhere [Supplementary Ref. 37].

Annexin-V-FITC/7AAD apoptosis assay.

Primary T-lymphocytes from mesenteric lymph nodes of *S.m.* infected mice were restimulated as described in section Material and Methods. Staurosporine and bile acids were dissolved in DMSO (staurosporine: 1 mM, UDCA: 50 mM and *nor*UDCA: 500 mM stock solution) and 24 h to restimulation added to the cell culture medium (final concentration staurosporine: 1 µM, UDCA: 50 µM and *nor*UDCA: 500 µM) and incubated for 4 hours. Annexin-V staining was performed using Annexin V Apoptosis Detection Kit FITC (affimetrix ebioscience, Frankfurt/Main, Germany). Five µl of 7-Aminoactinomycin D (7-AAD, life technologies, Darmstadt, Germany) was added following Annexin-V staining (AnV staining) and then analyzed using flow cytometry.

CFSE Cell proliferation assay.

To verify BrdU Assay CellTrace™ (Invitrogen Life Technologies, Darmstadt, Germany) was performed according to the manufacturer's instructions with 5 µM CellTrace™ reagent and following flow cytometry analysis after 48 hours.

RNA isolation, reverse-transcription and TaqMan real-Time polymerase chain reaction.

Liver specimens were snap-frozen in nitrogen-cooled methylene butane and then stored in liquid nitrogen until RNA analysis. Total RNA was isolated and reversed into DNA using the GeneAmp Gold RNA PCR Core Kit (Applied Biosystems, Vienna, Austria) according to the manufacturer's instructions. Real-time PCR (RT-PCR) for CIITA was performed in duplicate as described before [Supplementary Ref. 38]. RNA levels are normalized to 36b4 ribosomal gene expression levels. The following primers were used: CIITA: sense, 5'-CAAGTCCCTGAAGGATGTGGA-3'; anti-sense, 5'-ACGTCCATCACCCGGAGGGAC-3'; 36b4: sense, 5'-AGATGCAGCAGA-TCCGCA-3'; anti-sense, 5'-GTTCTTGCCCATCAGCACC-3'. 5'-Col1a2: sense, GCA-GGGTTCCAACGATGTTG-3'; anti-sense, 5'-GCAGCCATCGACTAGGACAGA-3'; IFN-gamma: sense, 5'-TCAAGTGGCATAGATGTGGAAGAA-3'; anti-sense, 5'-TGG-CTCTGCAGGATTTTCATG-3'; IL-1 beta: sense, 5'-CTGGTGTGTGACGTT-CCCATTA-3'; anti-sense, 5'-CCGACAGCACGAGGCTTT-3'; iNOS: sense, 5'-ACATCAGGTCGGCCATCACT-3'; anti-sense, 5'-CGTACCGGATGAGCTGTGAA-TT-3'; TNF-alpha: sense, GACCCTCACACTCAGATCATCTTCT; anti-sense, CCTCCACTTGGTGGTTTGCT; MMP-2: sense, 5'-CTTTGAGAAGGATGGCAA-GTATGG-3'; anti-sense, 5'-TTGTAGGAGGTGCCCTGGAA-3'; TIMP-1: sense, 5'-CATGGAAAGCCTCTGTGGATATG-3'; anti-sense, 5'-AAGCTGCAGGCATTGAT-GTG-3';TGF-beta1: sense, 5'-TGACGTCA-CTGGAGTTGTACGG-3'; anti-sense, 5'-

GGTTCATGTCATGGATGGTGC-3’. Primer for Arg1 (Mm00475988_ml) and Retnla (Mm00445109_ml) were purchased from Applied Biosystems (Foster City, CA).

Immunohistochemistry for F4/80, CD11b, CD3, MPO, Ki-67, K19 and alpha-SMA.

To quantify and characterize the hepatic inflammatory cell infiltrate immunohistochemistry (IHC) for F4/80$^+$, CD11b$^+$, CD3$^+$, MPO$^+$, Ki-67$^+$, K19$^+$ and alpha-SMA$^+$ cells was performed on formaldehyde (4% neutral-buffered) fixed, paraffin embedded liver sections or on cryosections using monoclonal mouse antibodies as described previously [Supplementary Ref. 38] (**Supplementary Table 1**). The number of positive cells was determined microscopically by counting positive cells in 20 hepatic granulomas (hg) per liver slide of each individual mouse (control: n(hg) = 140; UDCA: n(hg) = 240; *nor*UDCA: n(hg) = 280) under blinded conditions.

Western blotting for alpha-smooth muscle actin.

Liver tissue was homogenized using an ultrasonic probe. Homogenates (30 µg of protein) were run on a 10% sodium dodecyl sulphate polyacrylamide gel, transferred to nitrocellulose, and blotted with a monoclonal mouse antibody against alpha-SMA (dilution1:2000; DAKO). Binding was detected by using peroxidase-conjugated rabbit immunoglobulins against mouse and rat immunoglobulins (DAKO), and peroxidase activity was visualized by using an enhanced chemi-luminescence method described previously [Supplementary Ref. 38].

Supplementary figures and Supplementary figure legends

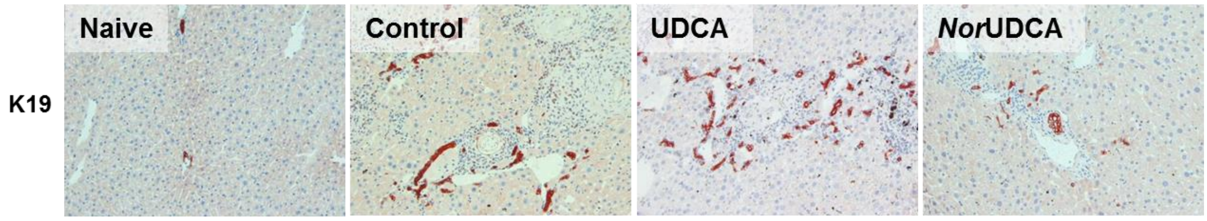

Supplementary Figure 1.

***Nor*UDCA but not UDCA reduces ductular proliferation.** IHC for K19 revealed ductular reaction around *S.m.* egg-granulomas in all infected groups. *Nor*UDCA reduced bile duct proliferation and K19 protein levels in contrast to UDCA with most apparent ductular reaction and K19 protein level.

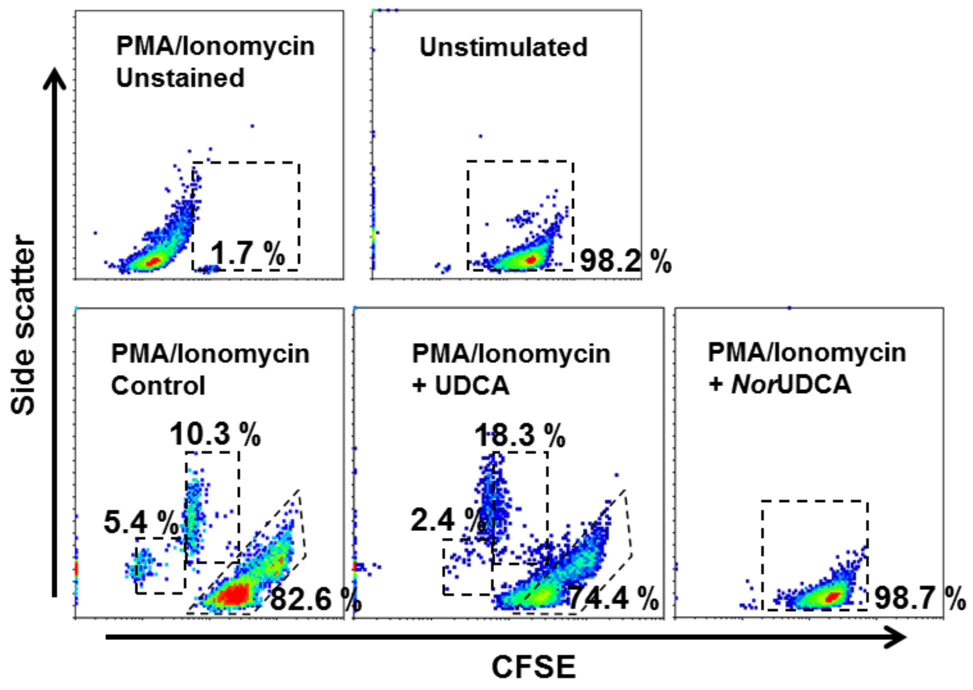

Supplementary Figure 2.

***Nor*UDCA but not UDCA blocks up T-lymphocyte restimulation.** To validate receptor dependent T-lymphocyte restimulation by CD3/CD28, isolated T-lymphocytes were restimulated in a T-cell-receptor independent manner using PMA/Ionomycin. Without incubation with bile acids (control) or after supplementation of UDCA (50 µM) T-lymphocytes start to proliferate as proven by reduction of intracellular CSFE content. *Nor*UDCA (500 µM) incubation led to a complete inhibition of T-lymphocyte restimulation. For validation of the method and reproducibility of the results the experiments were repeated in two independent series.

Supplementary Tables

Supplementary Table 1. Antibodies for Immunohistochemistry used in this study.

Antibody	clone	section
**MPO**	RB373, Serotec	paraffin
**CD3**	MCA1477, Thermoscientific	paraffin
**CD11b**	M1/70, ebioscience	paraffin
**F4/80**	BM8, ebioscience	paraffin
**Ki-67**	SolA15, ebioscience	cryo
**K19**	A53-B/A2, BioLegend	paraffin
**alpha-SMA**	1A4, Sigma Aldrich	paraffin

Supplementary Table 2. mRNA expression pattern under various experimental conditions (Sybr Green analysis).

Variable	Naïve (n=6)	Control (n=5)	UDCA (n=4)	*Nor*UDCA (n=6)
**IL-1 beta**	100±29	223±80*	120±96	81±21$^{\#}$
**IFN-gamma**	100±46	360±90*	200±200	230±50*$^{\#}$
**iNOS**	100±36	1008±405*	418±122*$^{\#}$	539±79*$^{\#}$
**TNF-alpha**	100±42	385±160*	123±28$^{\#}$	210±49*$^{\#\dagger}$
**TGF-beta1**	100±13	164±42*	129±15*	117±12$^{*\#}$
**Col1a2**	100±21	381±121*	210±52$^{*\#}$	262±72*
**MMP-2**	100±38	745±423*	207±67$^{*\#}$	431±126*
**TIMP-1**	100±61*	3299±1069*	941±234$^{*\#}$	1687±690$^{*\#\dagger}$
**Arg1**	100±4	95±3	97±4	96±3
**Retnla**	100±8*	124±2*	112±9$^{*\#}$	103±3$^{\#\dagger}$

Data are expressed as a percentage of naive animals; means ± SD. Naive: untreated, uninfected mice; Control: untreated, infected mice; UDCA: UDCA-fed, infected mice; *Nor*UDCA: *Nor*UDCA-fed, infected mice; IL-1 beta: interleukin-1 beta; IFN-gamma: interferon-gamma; iNOS: inducible nitric oxide synthase; TNF-alpha: tumor necrosis factor-alpha; TGF-beta: transforming growth factor-beta; Col1a2: type I collagen; MMP-2: matrix metalloproteinase-2; TIMP-1: tissue inhibitor of metalloproteinase; Arg1: liver arginase 1; Retnla: Resistin-like molecule alpha . P<0.05 (*vs. Naive; $^{\#}$vs. Control; †vs. UDCA).

Supplementary Table 3. Ratio of mRNA levels of major matrix degradation enzyme (MMP-2) and its inhibitor (TIMP-1) under various experimental conditions (Sybr Green analysis).

	Ratio TIMP-1/MMP-2
**Naïve**	1
**Control**	4.43*
**UDCA**	4.6*
***Nor*UDCA**	3.91*#†

Data are expressed as ratio of normalized values of mRNA expression level of matrixmetallo-proteinase-2 (MMP-2) and tissue inhibitor of metalloproteinase (TIMP-1). P<0.05 (*vs. Naïve; #vs. Control; †vs. UDCA).

Supplementary References

[36] Inaba K, Inaba M, Romani N, Aya H, Deguchi M, Ikehara S, et al. Generation of large numbers of dendritic cells from mouse bone marrow cultures supplemented with granulocyte/macrophage colony-stimulating factor. J Exp Med 1992;176:1693-1702.

[37] Duvall RH, DeWitt WB. An improved perfusion technique for recovering adult schistosomes from laboratory animals. Am J Trop Med Hyg 1967;16:483-486.

[38] Wagner M, Fickert P, Zollner G, Fuchsbichler A, Silbert D, Tsybrovskyy O, et al. Role of farnesoid X receptor in determining hepatic ABC transporter expression and liver injury in bile duct-ligated mice. Gastroenterology 2003;125:825-838.
